# LncRNAs LY86-AS1 and VIM-AS1 Distinguish Plasma Cell Leukemia Patients from Multiple Myeloma Patients

**DOI:** 10.3390/biomedicines9111637

**Published:** 2021-11-08

**Authors:** Romana Bútová, Petra Vychytilová-Faltejsková, Jana Gregorová, Lenka Radová, Martina Almáši, Renata Bezděková, Lucie Brožová, Jiří Jarkovský, Zdeňka Knechtová, Martin Štork, Luděk Pour, Sabina Ševčíková

**Affiliations:** 1 Babak Myeloma Group, Department of Pathophysiology, Faculty of Medicine, Masaryk University, 625 00 Brno, Czech Republic; romanabutova@gmail.com (R.B.); vychytilova.petra@seznam.cz (P.V.-F.); gregorovajana2@gmail.com (J.G.); 2 Department of Molecular Medicine, Central European Institute of Technology (CEITEC), Masaryk University, 625 00 Brno, Czech Republic; avodar@gmail.com; 3 Department of Clinical Hematology, University Hospital Brno, 625 00 Brno, Czech Republic; almasi.martina@fnbrno.cz (M.A.); bezdekova.renata@fnbrno.cz (R.B.); 4 Institute of Biostatistics and Analyses (IBA), Faculty of Medicine, Masaryk University, 625 00 Brno, Czech Republic; brozova@iba.muni.cz (L.B.); jarkovsky@iba.muni.cz (J.J.); 5 Clinic of Internal Medicine, Hematology and Oncology, University Hospital Brno, 625 00 Brno, Czech Republic; knechtova.zdenka@fnbrno.cz (Z.K.); Stork.Martin@fnbrno.cz (M.Š.); Pour.Ludek@fnbrno.cz (L.P.)

**Keywords:** multiple myeloma, plasma cell leukemia, long non-coding RNA, next-generation sequencing, biomarkers, disease progression

## Abstract

Long non-coding RNAs (lncRNAs) are functional RNAs longer than 200 nucleotides. Due to modern genomic techniques, the involvement of lncRNAs in tumorigenesis has been revealed; however, information concerning lncRNA interplay in multiple myeloma (MM) and plasma cell leukemia (PCL) is virtually absent. Herein, we aimed to identify the lncRNAs involved in MM to PCL progression. We investigated representative datasets of MM and PCL patients using next-generation sequencing. In total, 13 deregulated lncRNAs (*p* < 0.00025) were identified; four of them were chosen for further validation in an independent set of MM and PCL patients by RT-qPCR. The obtained results proved the significant downregulation of lymphocyte antigen antisense RNA 1 (LY86-AS1) and VIM antisense RNA 1 (VIM-AS1) in PCL compared to MM. Importantly, these two lncRNAs could be involved in the progression of MM into PCL; thus, they could serve as promising novel biomarkers of MM progression.

## 1. Introduction

Diseases characterized by clonal lymphoplasmacytic cell proliferation and high immunoglobulins production are known as plasma cell dyscrasias [1,2]; they range from premalignant disease (MGUS—monoclonal gammopathy of unknown significance) to aggressive multiple myeloma (MM) [3]. 

MM represents 10% of hematological malignancies [4], with an incidence rate of 4.8/100,000 in the Czech Republic [5] and global age-standardized incidence rates between 0.1/100,000 and 5.3/100,000 [6]. MM is characterized by the aberrant expansion of malignant clonal plasma cells (PCs) inside the bone marrow (BM) microenvironment and the presence of abnormal monoclonal proteins in the serum and/or urine [7]. 

The physiological differentiation of B cells with rearranging somatic DNA in B-cell progenitors via VDJ recombination [8], somatic hypermutation, and class switch recombination involves double-stranded DNA breaks. Some can fuse to other breaks in the genome that involve oncogenes, giving rise to a subclone of cells with a growth advantage that may lead to MGUS and, eventually, MM [9]. 

MM used to be diagnosed based on the so-called CRAB features (hypercalcemia, renal insufficiency, anemia, and bone lesions). The International Myeloma Working Group (IMWG) updated the diagnostic criteria of MM in 2014 by the addition of myeloma-defining events; thus, a MM diagnosis is now based on the presence of at least one myeloma-defining event and plasmacytoma or ≤10% of clonal PCs [10]. 

Plasma cell leukemia (PCL) is a rare but most aggressive form of plasma cell dyscrasias with an incidence rate of 0.057/100,000 in the Czech Republic [11] and 0.04/100,000 in Europe [12]. In 1974, the renowned American hematologist Dr. Kyle used the percentage and absolute count of circulating PCs (cPCs) for the intuitive determination of a PCL diagnosis [13]. cPCs have a capability of entering the peripheral blood because of their independence from the BM microenvironment caused by the presence of further molecular aberrations and the diverse expression of chemokine receptors and adhesion molecules promoting tumor growth outside of the BM, the inhibition of apoptosis, and escape from the immune system [14]. 

Today, the presence of more than 20% of cPCs and their absolute count greater than 2 × 10^9^ per liter in the peripheral blood are the accustomed criteria for a PCL diagnosis [15]. However, many patients do not meet the absolute count criteria because of leukopenia or a poor BM reserve as the result of intensive treatment, chemotherapy, malnutrition, or myelodysplasia [15,16]. Thus, an update was suggested by the IMWG and WHO, proposing that only percentage criteria should be required for the disease diagnosis [17]. It was demonstrated that the presence of ≥5% cPCs in MM patients has a similar adverse prognostic impact on PCL patients, indicating that a lower threshold for the definition of PCL should be adapted in the future [18,19]. 

Based on the presence of previously diagnosed MM, PCL may be divided into two groups: primary PCL (pPCL) and secondary PCL (sPCL). pPCL patients have no history of previously diagnosed MM, and they are approximately 10 years younger than sPCL patients, with the highest incidence between 52 and 65 years. Despite a high sensitivity to chemotherapy, an aggressive clinical course with short remissions and reduced overall survival (OS) is present [20,21]. The median OS is under 1 year for pPCL patients receiving conventional chemotherapy [22,23,24]. A prolonged OS was described in patients who received lenalidomide–dexamethasone induction and underwent transplantation (median 28 months) [24]. On the other hand, sPCL represents a leukemic transformation of refractory MM. It is diagnosed in 1.8–4% of MM patients [25] for a median time of progression of 20–22 months [26]. However, these patients usually fail the treatment regimens [14], and their median OS is approximately 6–11 months [27]. 

Long non-coding RNAs (lncRNAs) are non-coding RNA molecules longer than 200 nucleotides (nt) that lack a protein-coding capacity [28]. They are key regulators of mRNA decay, alternative splicing, nuclear import, embryonic stem cell pluripotency, and other biological processes in eukaryotic cells [29,30,31,32]. LncRNAs can function at the transcriptional, post-transcriptional, and chromatin modification levels. Based on their structures, they operate as guides, signals, decoys, or scaffolds [33,34]. The deregulated expression of lncRNAs has been described in neurodevelopmental diseases and cancers [34]. LncRNAs may be clinically relevant, as they function as either oncogenes or tumor suppressors in hematopoietic development [35]. So far, several different lncRNAs have been implicated in MM pathogenesis [36,37,38,39], including initiation and progression in MM and PCL [40]. 

A recent study by Carrasco-Leon et al. [41] described the impact of lncRNAs on the behavior of MM, as their transcriptional dynamics approach revealed an exclusive expression of 989 lncRNAs in MM, from which 89 lncRNAs were *de novo* epigenomically activated. The attribution of lncRNA expression profiles to the survival predictions of MM patients was discussed in the study by Zhou et al. [42], who developed a lncRNA focus risk model. Shen et al. [43] investigated deregulated lncRNAs in MM and revealed the correlation between the high expression of LAMA5AS1 and the better prognosis of MM patients. Interestingly, Ronchetti et al. [37] developed a comprehensive catalog of deregulated lncRNAs in MM and listed lncRNAs with proximal genes to suggest a possibility of cis-regulatory relationships involved in MM pathogenesis.

As previous reports indicated the involvement of lncRNAs in the pathogenesis of plasma cell dyscrasias, the aim of this study was to compare the expression profiles of these molecules in the BM plasma cells (BMPCs) of MM and PCL patients in a two-phase biomarker study using a high-throughput approach, including next-generation sequencing (NGS) and subsequent RT-qPCR validation. Further, the expression of selected lncRNAs was correlated with the clinical–pathological data of patients. To the best of our knowledge, no similar study has been previously published.

## 2. Materials and Methods

### 2.1. Patient Characteristics and Sample Preparations 

Thirty-four MM BMPC samples and twenty-three PCL (12 pPCL and 11 sPCL) BMPC samples were obtained for this study. Based on the previous studies [18,19], the cut-off for PCL was set at 5% of the cPCs. The diagnosis was confirmed by a histopathological examination, biopsy, and flowcytometry. All the patients were diagnosed at the University Hospital Brno between the years 2000 and 2020 and signed an informed consent form approved by the ethics committee of the hospital in accordance with the current version of the Helsinki Declaration. The clinical characteristics of the patients, as well as previous therapies of sPCL patients, are summarized in Tables S1 and S2. 

The BMPCs were enriched by anti-CD138+ immunomagnetic beads and magnetic activated cell sorting with AutoMACS (MiltenyiBiotec) was used for their separation with ≥90% purity as previously described [44]. The samples were stored at −80 °C and thawed only once. 

#### 2.1.1. RNA Isolation

The total RNA was extracted from frozen-dry CD138+ pellets using the miRNeasy Mini kit (Qiagen, Germany) according to the manufacturer’s instructions. The concentration of isolated RNA was determined using the fluorometric quantification on Qubit 4.0 with the RNA Broad Range Assay kit (Thermo Fischer Scientific, MA, USA), and the quality of the extracted RNA was verified with RNA Integrity Number (RIN) determination using Broad Range RNA Screen Tape and Buffer on 2200 TapeStation (all from Agilent Technologies, CA, USA). 

#### 2.1.2. Next-Generation Sequencing

For NGS, 11 PCL samples and 6 MM samples were used. Ribosomal RNA (rRNA) was depleted using the Ribo-Zero-Gold rRNA Removal kit (Illumina, CA, USA) according to the manufacturer´s recommendations with 1000-ng input of RNA. rRNA-depleted RNA was quantified using the RNA High Sensitivity Assay kit on Qubit 4.0 (Qiagen, Germany). The successful depletion of rRNA was verified on 2200 Tape Station, performing agarose electrophoresis with High Sensitivity RNA Screen Tape and Buffer (all from Agilent Technologies, CA, USA). Subsequently, the cDNA libraries were prepared from rRNA-depleted samples using the NEBNext Ultra II Directional RNA Library Prep kit and NEBNext Multiplex Oligos (Dual Index Primers Set 1) (all from Illumina, CA, USA). The concentrations of the prepared cDNA libraries were determined on Qubit 4.0 using the dsDNA High Sensitivity Assay kit (Qiagen, Germany), and the cDNA library fragment lengths were determined on 2200 TapeStation using High Sensitivity D1000 Screen Tape and Buffer (all from Agilent Technologies, CA, USA) [45]. Finally, the paired-end sequencing (2 × 75 nucleotides) of 2.1-pM libraries was performed on the NextSeq 500 Sequencing System using the NextSeq 500/550 High Output kit v.2 (all from Illumina, CA, USA). 

#### 2.1.3. RT-qPCR Validation

Validation of the NGS results was performed on 28 MM samples and 12 PCL samples using RT-qPCR. The isolated RNA was reverse-transcribed using the High-Capacity cDNA Reverse Transcription kit from Thermo Fisher Scientific (MA, USA) to synthesize cDNA from 1000 ng of RNA. An analysis of four significantly deregulated lncRNAs was performed using the TaqMan™ Gene Expression Master Mix (Thermo Fisher Scientific, CA, USA) and individual TaqMan assays for the selected lncRNAs (Life Technologies, Table S3) on the QuantStudio™ 3 Real-Time PCR System (Thermo Fisher Scientific, CA, USA). 

#### 2.1.4. Statistical Analysis

Quality control of the sequencing data (RNA-seq reads) was performed using fastqc. The sequences were mapped on the human reference genome GRCh38 using the STAR aligner (version 2.2.1) [46]. Further analyses were performed using the R/Bioconductor packages [47]. The GENCODE database of lncRNAs version 32 was used for feature counts by applying the Rsubread package procedure [48]. The filtered read counts were pre-normalized by adding normalization factors within the edgeR package and further between-sample normalized by the voom function in the limma package [49,50,51]. After the determination of the normalized expression levels, the differentially expressed lncRNAs between the PCL and MM patients were screened by applying block linear model fitting and a Bayes approach from the limma package. The obtained *p*-values were adjusted for multiple testing using the Benjamini–Hochberg method. 

The RT-qPCR data were analyzed using the relative quantification approach 2^−ΔCt^ in which the ΔCT was calculated as follows: ΔCT = CT_targetlncRNA_ − CT_PPIA_. PPIA was chosen as a housekeeping gene for normalization using the TATAA Reference Gene Panel (TATTA Biocenter, Göteborg, Sweden) and the GeNorm and NormFinder software programs of GenEx (bioMCC, Freising, Germany) on the selected 9 MM and 9 PCL samples, with PPIA as the most stable across the samples (Figure S1). The associations of two categorical/categorical and continuous variables were tested using the two-sided nonparametric Mann–Whitney *U* test. The correlation of two continuous variables was assessed using two-sided Spearman’s correlation (SPSS software, IBM Corp. Released 2017, Version 25.0). *p*-values of less than 0.05 were considered statistically significant. The sensitivity and specificity were calculated using the receiver operating characteristic (ROC) analysis. 

## 3. Results

### 3.1. Deregulation of lncRNAs in the Exploration Phase

**Figure 1:**
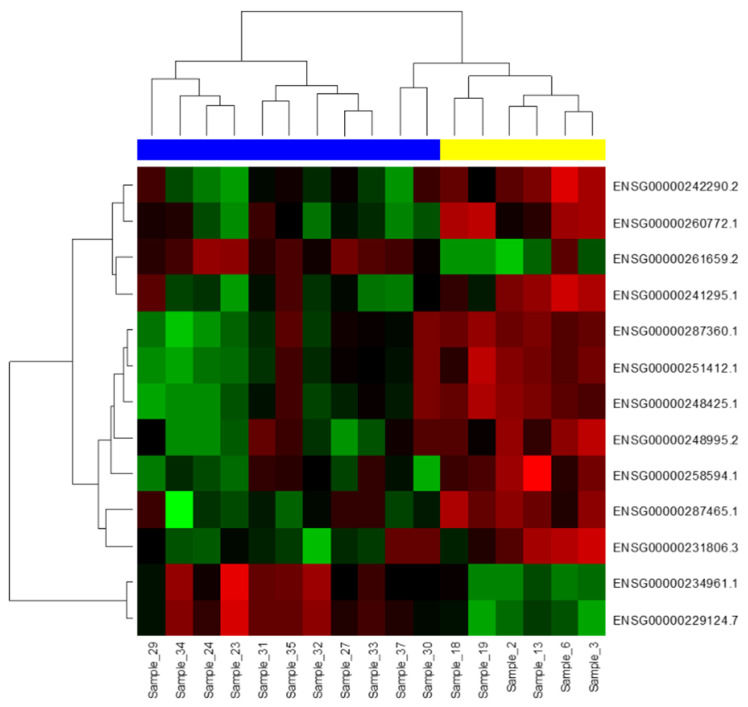
Hierarchical clustering and heatmap visualizing 13 lncRNAs differentially expressed between six multiple myeloma (MM) samples (yellow) and eleven plasma cell leukemia (PCL) samples (blue) (*p* ≤ 0.00025) in the exploration phase of the study. Red represents high expression levels, and green represents low expression levels.

NGS was performed on 11 PCL and 6 MM BMPCs samples to identify the lncRNA expression patterns. The sequenced samples contained, on average, 58.686.298 ± 11.700.608 reads, and the proportion of pure reads mapped to the genome was > 98%. The sequencing Q30 of each sample was higher than 93%, indicating a good base quality for the downstream analyses. The sequencing output of the prepared libraries resulted in 17,910 different lncRNAs. A further analysis involved 7895 lncRNAs with more than one read per million in all the analyzed samples. In total, 52 different lncRNAs were found to be significantly deregulated between the MM and PCL samples with an adjusted *p* < 0.001 (Table S4). From those, 13 lncRNAs were observed to be deregulated between these two groups with an adjusted *p*-value ≤ 0.00025 (Figure 1).

### 3.2. Validation of Selected lncRNAs Deregulated between MM and PCL Samples

For further validation on a larger cohort of the 28 MM and 12 PCL samples, RT-qPCR was performed, analyzing the expression of four candidate lncRNAs: lymphocyte antigen antisense RNA 1 (LY86-AS1), MIR9-3 Host Gene (MIR9-3HG), VIM antisense RNA 1 (VIM-AS1), and Prostate Cancer-Associated Transcript 7 (PCAT7). The selection of the validated lncRNAs was based on the statistical significance (*adj p* < 0.001), fold change level (|logFC| > 1.5), their average expression (average log expression values > 3), and previous data from published papers. 

**Figure 2:**
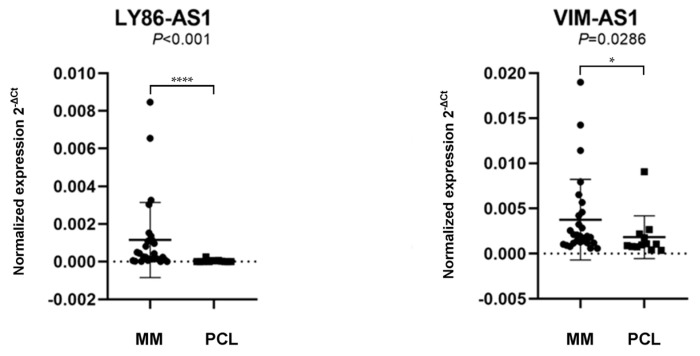
Comparison of the expression levels of lncRNA lymphocyte antigen antisense RNA 1 (LY86-AS1) and VIM antisense RNA 1 (VIM-AS1) in patients with multiple myeloma (MM) and plasma cell leukemia (PCL). The lncRNAs levels in a validation cohort of patient samples, with the data presentation as the median value of the normalized lncRNA expression and interquartile range (25,75) with the standard deviation. The values were compared using the Mann–Whitney *U*-test. The expression levels were defined as the 2^−ΔCt^ values normalized to the PPIA expression levels.* *p* < 0.05, **** *p* < 0.0001.

The results revealed that LY86-AS1 (*p* < 0.001) and VIM-AS1 (*p* = 0.0286) are significantly upregulated in MM patients compared to PCL patients (Figure 2 and Table S5). As the differences in the expression levels of MIR9-3HG (*p* = 0.1965) and PCAT7 (*p* = 0.3763) between the MM and PCL samples were not statistically significant, they were excluded from any further analyses. 

### 3.3. ROC Analyses of lncRNAs LY86-AS1 and VIM-AS1

**Figure 3:**
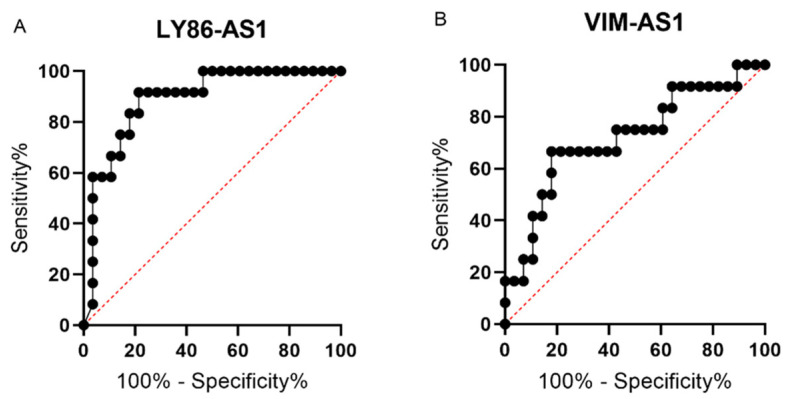
Receiver operating characteristic (ROC) curves for lncRNAs LY86-AS1 (**A**) and VIM-AS1 (**B**) that were found to be significantly deregulated in bone marrow plasma cells (BMPCs) of multiple myeloma (MM) and plasma cell leukemia (PCL) patients in the validation phase of the study.

A ROC curve analysis was performed to identify the optimal cut-off values for two deregulated lncRNAs by the maximization of the sum of the sensitivity and specificity. For LY86-AS1, the ROC analysis showed an ability to distinguish MM patients from PCL patients with a sensitivity of 92% and specificity of 79%, with the area under the curve (AUC) = 0.8884 using the cut-off value of ≤0.0000812 (*p* < 0.0001) (Figure 3A). In the case of VIM-AS1, the ROC analysis showed a sensitivity of 67%, specificity of 82%, and AUC = 0.7202, with a cut-off value of ≤ 0.00112 (*p* < 0.05) (Figure 3B). Unfortunately, the combined analysis of both lncRNAs did not lead to an increased sensitivity or specificity. These results indicate the potential diagnostic value of identified lncRNAs and imply the possibility of improved discrimination between the MM and PCL diagnoses.

### 3.4. Expression of lncRNA VIM-AS1 Is Associated with the Light Chain Characteristics in PCL Patients

A Spearman bivariate correlation was performed with continuous quantities to assess the correlation between the lncRNA expression levels and clinical parameters of the patients. In the MM patients, LY86-AS1 showed a trend of negative correlation with the thrombocyte count (r = −0.323), but the correlation was not significant (*p* = 0.094). A comparison between the lncRNA relative quantification and baseline characteristics was performed using the Mann–Whitney *U* test with categorical variables. It revealed the correlation of the VIM-AS1 levels with kappa light chain (*p* = 0.048). The correlations with the other clinical–pathological data of the patients, as well as the results of the cytogenetics, were not statistically significant.

## 4. Discussion

Non-coding RNA molecules were considered genetically accumulated waste. However, as new techniques were introduced into the research, it was discovered that up to 90% of the total genome sequences are transcriptionally active, but only 2% are represented by protein-coding genes [29]. Soon after, lncRNAs became the subject of intensive translational research, especially for their participation in key cellular actions and functions [52]. Thus, there was no surprise that they are involved in the tumorigenesis of many malignant diseases [53,54,55], including plasma cell dyscrasias [56]. Both PCL and MM have been systematically studied for the deregulated expression of lncRNA-based biomarkers, such as markers of prognostic estimations, predictive evaluations of treatment effectiveness or disease diagnosis, stratification, and classification [57].

Interestingly, PCL patients with lower levels of cPCs have the same poor prognosis, which challenges the disease definition. The topic on the definition of PCL and the distinction between PCL and MM is currently being discussed in the international myeloma community. The first diagnostic criteria for PCL were established in 1974 by Noel P. and Kyle R.A. as the presence of an absolute plasma cell count more than 2 × 10^9^/L and >20% of the total white blood count. These criteria are still followed by the WHO [58,59]. The passing criteria (before 1974) did not consider the investigation of PC clonality, as the first primitive electric cell sorting device was reported only up to 1965 [60]. It is important to distinguish the cause of reactive plasmacytosis by PC phenotype determination, as it can mimic PCL in patients with a variety of infections (such as Staphylococcal sepsis) or neoplastic conditions [61,62].

Suggestions for updated diagnostic, response, and progression criteria with an adjusted clonality assessment by flow cytometry for the diagnosis of PCL were discussed by the IMWG in 2013, especially in relation to the minimalization of risk of early death with onset therapy induction [14]. Bezdekova et al. [63] discussed a need to determine the flow cytometry criteria, which reduces the original cut-off to at least 10%, as the number of cPCs is correlated with a shorter OS. Similarly, Granell et al. [18] described the impact of cPCs on the survival of patients with MM and stated that the presence of ≥ 5% cPCs in these patients has a similar prognostic impact as PCL. Therefore, we used the same cut-off for our PCL criteria.

Using NGS, we determined 52 lncRNAs with significantly different expressions between the MM and PCL BMPC samples, for which the diagnostic criteria were set up at the presence of more than 5% of cPCs in the peripheral blood. Four lncRNAs (VIM-AS1, MIR9-3HG, LY86-AS1, and PCAT7) were chosen for the validation phase on the independent set of BMPC samples from the MM and PCL patients. LncRNA LY86-AS1 and lncRNA VIM-AS1 were significantly upregulated in the MM patients in comparison to the PCL patients (*p* ≤ 0.05) The subsequent ROC revealed that it was possible to distinguish between the MM and PCL patients with a high sensitivity and specificity for both lncRNAs.

When evaluating the cohort of MM and PCL patients, VIM-AS1 was found to be negatively correlated with kappa light chains in the PCL patients. The expression levels of the chosen lncRNAs were not significantly correlated with the available biochemical parameters, but there was a trend of a negative correlation between lncRNA LY86-AS1 and the thrombocyte count in the MM patients. Thrombocytopenia is a common sign of MM [64]. In the study by Li et al. [65], a relation between autoimmune idiopathic thrombocytopenic purpura and an increased level of lncRNA was described. It is suggested that lncRNAs affect and mediate the functions of the gene network in this disease [65].

There are currently no known studies describing the correlation between the deregulated level of lncRNA expressions and MM or PCL, and therefore, their significance in the pathogenesis processes of these diseases remains unclear. The decreased expression of lncRNA LY86-AS1 was described in type 2 diabetes mellitus patients compared to a healthy control group [66] and was identified as a gender-associated lncRNA negatively correlated with the Braak stage of Alzheimer´s disease [67]. There is also evidence of LY86-AS1 involvement in various cancers, such as central nervous system cancer, breast carcinoma, metastatic melanoma, astrocytoma, and lung adenocarcinoma [68]. Interestingly, polymorphism rs12192707 in this lncRNA was associated with changes in the immune system response in pemphigus foliaceus patients [69], and similar effects could be found also in MM patients. Nevertheless, further studies are needed. The deregulated expression of lncRNA VIM-AS1 was described in type 2 diabetes mellitus [70], in gastric cancer, with a role in activating the Wnt/β-catenin pathway [71], prostate cancer [72], breast cancer [73], and many others, indicating its role in the pathogenesis of numerous diseases. Importantly, the sponging of miR-29, a key miRNA in hematological malignancies [74], by VIM-AS1 was confirmed in diabetic retinopathy [75]. However, deeper knowledge of its function and contribution to MM initiation and progression is still missing.

One of the subjects of possible future research following this work remains the correlation of the acquired information with the survival of MM and PCL patients in order to interpret the prognostic potential of LY86-AS1 and VIM-AS1. However, the limitations of this study included the relatively small sample size caused by the low incidence of PCL; therefore, additional laboratory and clinical studies on a larger cohort of patients could contribute to a better understanding of the roles of lncRNAs in the pathogenesis of MM and PCL.

## 5. Conclusions

In this study, we aimed to investigate if lncRNA molecules are deregulated between MM and PCL patients. We showed that lncRNAs LY86-AS1 and VIM-AS1 are significantly upregulated in a MM compared to a PCL diagnosis and might be involved in the progression of the disease. Nevertheless, the exact mechanisms remain unclear.

## Data Availability

The NGS data will be downloaded into a publicly available database.

## Supplementary Materials

The following are available online at https://www.mdpi.com/article/10.3390/biomedicines9111637/s1: Table S1: Clinical–pathological data of multiple myeloma (MM) and plasma cell leukemia (PCL) patients used in a NGS and RT-qPCR analysis. Table S2: Previous therapy in any treatment line before a sPCL diagnosis. Table S3: TaqMan assays of validated long non-coding RNAs (lncRNAs). Table S4: LncRNAs with significantly deregulated expression levels with *p* < 0.001 detected by next-generation sequencing (NGS). Table S5: Normalized expression levels of significantly deregulated lncRNAs LY86-AS1 and VIM-AS1 in the validation phase of the study. Figure S1: Housekeeping gene analysis for normalization using the TATAA Reference Gene Panel.
