# Supplementary material for: LncRNAs LY86-AS1 and VIM-AS1 Distinguish Plasma Cell Leukemia Patients from Multiple Myeloma Patients
Source: Biomedicines. 2021 Nov 8;9(11):1637. doi: 10.3390/biomedicines9111637 (PMC8615960; doi:10.3390/biomedicines9111637)
Supplement: Supplementary file 1 [file biomedicines-09-01637-s001.zip › biomedicines-1414265-supplementary.pdf]

biomedicines

MDPI

SUPPLEMENTARY FIGURES AND TABLES

Table S1. Baseline characteristics of multiple myeloma (MM) and plasma cell leukemia (PCL) patients used in next-generation sequencing (NGS) and RT-qPCR analysis.

**Baseline characteristics[1]:**	**MM**	**PCL**
No. of patients	34	23
Gender: males - females	29.4 - 70.6	44.2 - 55.8
Age median (min-max) (years)	72 (45- 86)	69 (42 - 83)
ISS stage: I-II-III	17.6 - 41.2 - 41.2	NA – 34.1 - 65.9
ECOG PS: 0-1-2-3-4	14.7 - 35.3 - 32.4 -11.8 - 5.9	43.8 - 39.6 - NA - NA
Ig isotype: G-A-LC only-M-D-Bicl-NS	64.7 - 17.6 - 14.7 - NA – NA - NA - 2.9	40.0 - 17.5 - 19.2 - 9.1 – NA - NA - NA
Light chains: kappa-lambda	72.7 - 27.3	56.8 - 43.2
Previous treatment lines: 0-1-2-4	NA	50 – 30 – 10 - 10
cPCs (%)	0.2 (0.00 – 4.6)	19.4 (7.0 – 67.5)
**Biochemistry at diagnosis[2]:**		
Hemoglobin level (g/l)	95.7 (63.2 - 141.0)	93.2 (66.9 - 139.0)
Thrombocyte count (x10^9/l)	182.0 (69.4 - 357.0)	83.6 (37.6 - 201.0)
Calcium total level (mmol/l)	2.4 (2.0 - 3.5)	2.5 (1.9 - 4.7)
Albumin level (g/l)	32.5 (19.2 - 46.5)	34.4 (22.5 – 50.0)
Creatinine level (µmol/l)	92.0 (50.0 - 913.0)	164.5 (49.0 - 781.0)
β2-microglobulin (mg/l)	4.4 (2.1 - 50.0)	8.7 (2.4 - 44.9)
Lactate dehydrogenase (µkat/l)	3.6 (1.4 - 7.3)	6.0 (1.2 – 22.9)
C-reactive protein (mg/l)	4.2 (0.0 - 69.2)	10.7 (1.0 - 42.3)
**Cytogenetics at MM / PCL diagnosis[3]:**		
No. of patients	34 (100%)	12 (52%)
IgH disruption: positive	30.80%	100.00%
Translocation t(11;14): positive	0.00%	100.00%
Translocation t(4;14): positive	31.30%	60.00%
Translocation t(6;14): positive	0.00%	100.00%
Translocation t(14;16): positive	6.70%	33.30%
del(13)(q14)/monosomy 13: positive	76.90%	63.60%
gain 1q21: positive	65.40%	90.90%
del(17)(p13): positive	20.00%	27.30%
Hyperdiploidy: positive	33.30%	37.50%

[1]: Described using N (%) and/ or median (minimum - maximum). [2]: Described using median (minimum - maximum). [3]: Described using N(%). Only 12 samples from PCL patients out of 23 have been analyzed for cytogenetic changes. NA = not available, Bicl – biclonal, NS – non-secretory

Table S2: Previous therapy in any treatment line before sPCL diagnosis.

**Previous therapy of patients diagnosed with sPCL (N = 11 patients with sPCL)**[1]	**Statistics**[2]
**Proteasome inhibitors (PI)**	**8 (72.7%)**
bortezomib	8 (72.7%)
**Immunomodulatory drugs (IMiD)**	**6 (54.5%)**
thalidomide	4 (36.4%)
lenalidomide	3 (27.3%)
**PI + IMiD**	**5 (45.5%)**
**Corticosteroids**	**9 (81.8%)**
**Conventional chemotherapy**	**8 (72.7%)**
**Transplantation**	**3 (27.3%)**
**Number of previous treatment lines**	
1	6 (54.5%)
2	3 (27.3%)
4	2 (18.2%)
median (min–max)	1 (1–4)
**Time from MM to PCL diagnosis (months),** median (min–max)	14.0 (3.8–60.1)

[1]Respective treatment modalities could be combined.

[2]Described using N (%) in categorical variables and median (minimum–maximum) in continuous variables.

Table S3: TaqMan Assays of validated long non-coding RNAs (lncRNAs).

**LncRNAs**	**Life Technologies Assay ID**	**Catalog Number**
LY86-AS1	Hs00543584_m1	4331182
VIM-AS1	Hs04402632_m1	4426961
MIR9-3HG	Hs00288663_m1	4331182
PCAT7	Hs04406635_m1	4426961

Table S4: LncRNAs with significantly deregulated expression level between multiple myeloma patients and patients with plasma cell leukemia (adjusted $P < 0.001$) as detected by next-generation sequencing analysis during the exploration phase of the study.

LncRNA	ENSG[1]	logFC[2]	AveExpr[3]	*P*-value	adj *P*-value
VIM-AS1	ENSG00000229124	1.597	11.059	2.669×10^{-9}	2.034×10^{-5}
AL358053.1	ENSG00000287465	-2.309	3.481	9.307×10^{-8}	1.489×10^{-4}
ZBTB20-AS5	ENSG00000242290	-1.417	6.183	1.038×10^{-7}	1.489×10^{-4}
AL133415.1	ENSG00000234961	1.303	11.585	1.021×10^{-7}	1.489×10^{-4}
AC006296.2	ENSG00000251412	-2.804	1.693	1.087×10^{-7}	1.489×10^{-4}
AC006296.1	ENSG00000248425	-2.708	1.173	1.173×10^{-7}	1.489×10^{-4}
PCAT7	ENSG00000231806	-3.876	3.370	2.308×10^{-7}	1.984×10^{-4}
LINC02231	ENSG00000248995	-2.439	0.964	2.124×10^{-7}	1.984×10^{-4}
AC012321.1	ENSG00000260772	-1.060	5.570	2.727×10^{-7}	1.984×10^{-4}
AC006296.3	ENSG00000287360	-2.470	1.931	2.709×10^{-7}	1.984×10^{-4}
Z92544.1	ENSG00000261659	1.089	5.126	2.996×10^{-7}	1.984×10^{-4}
AC021979.1	ENSG00000258594	-2.874	1.777	3.126×10^{-7}	1.984×10^{-4}
ZBTB20-AS2	ENSG00000241295	-1.553	4.700	3.682×10^{-7}	2.158×10^{-4}
AC079416.4	ENSG00000280227	-1.000	5.015	5.556×10^{-7}	2.919×10^{-4}
LINC02576	ENSG00000232613	-2.142	5.227	5.748×10^{-7}	2.919×10^{-4}
AL391834.2	ENSG00000273226	-1.267	3.945	6.165×10^{-7}	2.936×10^{-4}
AC087752.3	ENSG00000253878	-1.557	5.359	7.384×10^{-7}	3.309×10^{-4}
AL162719.1	ENSG00000285717	-2.995	2.160	8.139×10^{-7}	3.445×10^{-4}
AC012313.1	ENSG00000232098	1.230	4.096	9.237×10^{-7}	3.520×10^{-4}
AC092155.1	ENSG00000226622	-2.470	2.229	9.642×10^{-7}	3.520×10^{-4}
AC119673.2	ENSG00000286619	-3.273	4.409	1.013×10^{-6}	3.520×10^{-4}
ZBTB20-AS1	ENSG00000241560	-1.199	7.417	1.016×10^{-6}	3.520×10^{-4}
AL450992.1	ENSG00000229021	2.448	2.044	1.218×10^{-6}	4.035×10^{-4}
AC009948.3	ENSG00000279598	-1.988	5.019	1.716×10^{-6}	5.231×10^{-4}
AC093010.2	ENSG00000259976	-1.312	10.739	1.646×10^{-6}	5.226×10^{-4}
LINC01712	ENSG00000231364	-2.378	1.993	1.909×10^{-6}	5.595×10^{-4}
AC068051.1	ENSG00000197585	-1.969	3.451	2.330×10^{-6}	6.335×10^{-4}
AL161640.1	ENSG00000230404	-3.135	1.409	2.439×10^{-6}	6.335×10^{-4}
AC008569.1	ENSG00000267379	1.212	6.403	2.425×10^{-6}	6.335×10^{-4}
SEMA6A-AS1	ENSG00000248445	-1.068	4.589	2.520×10^{-6}	6.335×10^{-4}
AL024508.1	ENSG00000272189	-1.610	2.343	2.614×10^{-6}	6.335×10^{-4}
AC016745.2	ENSG00000272563	-2.614	3.020	2.744×10^{-6}	6.335×10^{-4}
AL360181.2	ENSG00000235245	1.543	2.792	2.664×10^{-6}	6.335×10^{-4}
AC026333.4	ENSG00000274191	-2.021	2.220	3.656×10^{-6}	8.193×10^{-4}
AP003721.1	ENSG00000256196	1.575	2.802	4.681×10^{-6}	9.412×10^{-4}
PLCB1-IT1	ENSG00000225479	2.520	3.080	4.739×10^{-6}	9.412×10^{-4}
LY86-AS1	ENSG00000216863	-2.644	4.628	4.698×10^{-6}	9.412×10^{-4}
AC068491.2	ENSG00000270190	2.642	1.366	4.567×10^{-6}	9.412×10^{-4}
AC079906.1	ENSG00000274723	-2.188	1.122	5.136×10^{-6}	9.412×10^{-4}
AC108463.3	ENSG00000271590	2.628	2.792	5.261×10^{-6}	9.412×10^{-4}
AL022098.1	ENSG00000287920	-1.251	7.062	5.066×10^{-6}	9.412×10^{-4}
MIR9-3HG	ENSG00000255571	-4.075	5.022	5.463×10^{-6}	9.459×10^{-4}
LINC02090	ENSG00000230969	-2.330	3.018	5.747×10^{-6}	9.596×10^{-4}
AP000560.1	ENSG00000278879	-1.135	7.303	5.245×10^{-6}	9.412×10^{-4}

SEMA6A-AS2	ENSG00000249167	-2.223	1.862	$5.822x10^{-6}$	$9.596x10^{-4}$
IFNG-AS1	ENSG00000255733	-1.835	8.785	$5.312x10^{-6}$	$9.412x10^{-4}$
LINC02389	ENSG00000255693	-2.680	3.853	$6.172x10^{-6}$	$9.596x10^{-4}$
AL158206.1	ENSG00000260912	-1.689	4.848	$5.987x10^{-6}$	$9.596x10^{-4}$
LINC00373	ENSG00000231019	-2.223	2.116	$6.589x10^{-6}$	$9.982x10^{-4}$
AC002350.2	ENSG00000278993	1.043	4.097	$6.797x10^{-6}$	$9.982x10^{-4}$
AC010680.3	ENSG00000271011	-1.897	2.232	$6.813x10^{-6}$	$9.982x10^{-4}$
AP005212.4	ENSG00000283294	-2.081	6.930	$6.156x10^{-6}$	$9.596x10^{-4}$

[1] ENSG – Ensembl Gene ID, [2] FC - fold change, [3] AveExpr - average log-expression values.

Table S5: Normalized expression levels of significantly deregulated lncRNAs lymphocyte antigen antisense RNA 1 (LY86-AS1) and VIM Antisense RNA 1 (VIM-AS1) in validation phase of the study.

Diagnosis	Sample	LY86-AS1 ($2^{-\Delta Ct}$)	VIM-AS1 ($2^{-\Delta Ct}$)
MM	1	$5.143x10^{-5}$	$1.185x10^{-3}$
	2	$1.440x10^{-4}$	$1.141x10^{-2}$
	3	$8.461x10^{-3}$	$1.899x10^{-2}$
	4	$3.038x10^{-3}$	$7.941x10^{-3}$
	5	$1.992x10^{-5}$	$6.531x10^{-3}$
	6	$1.636x10^{-4}$	$1.921x10^{-3}$
	7	$1.371x10^{-4}$	$2.107x10^{-3}$
	8	$1.083x10^{-3}$	$1.423x10^{-2}$
	9	$1.358x10^{-3}$	$1.151x10^{-3}$
	10	$2.324x10^{-4}$	$4.604x10^{-3}$
	11	$6.596x10^{-5}$	$2.852x10^{-3}$
	12	$8.806x10^{-6}$	$1.924x10^{-3}$
	13	$2.585x10^{-4}$	$5.676x10^{-3}$
	14	$3.262x10^{-3}$	$1.414x10^{-3}$
	15	$9.312x10^{-5}$	$5.884x10^{-4}$
	16	$2.485x10^{-5}$	$4.208x10^{-3}$
	17	$2.383x10^{-4}$	$1.027x10^{-3}$
	18	$4.481x10^{-4}$	$1.282x10^{-3}$
	19	$6.547x10^{-3}$	$3.235x10^{-3}$
	20	0	$1.717x10^{-3}$
	21	$9.621x10^{-4}$	$1.190x10^{-3}$
	22	$4.261x10^{-4}$	$1.399x10^{-3}$
	23	$8.118x10^{-4}$	$2.557x10^{-3}$
	24	$4.998x10^{-4}$	$5.743x10^{-4}$
	25	$1.130x10^{-3}$	$2.071x10^{-3}$
	26	$1.524x10^{-3}$	$1.798x10^{-3}$
	27	$1.211x10^{-4}$	$9.055x10^{-4}$
	28	$1.136x10^{-3}$	$7.818x10^{-4}$
PCL	1	$6.264x10^{-6}$	$8.823x10^{-4}$
	2	$7.168x10^{-6}$	$7.842x10^{-4}$
	3	0	$3.644x10^{-4}$
	4	$2.259x10^{-5}$	$1.087x10^{-3}$
	5	$3.463x10^{-6}$	$2.650x10^{-3}$
	6	$6.934x10^{-5}$	$2.174x10^{-3}$
	7	$2.530x10^{-4}$	$1.047x10^{-3}$
	8	$4.212x10^{-5}$	$3.985x10^{-4}$
	9	$5.282x10^{-5}$	$9.732x10^{-4}$
	10	$1.990x10^{-6}$	$1.766x10^{-3}$
	11	$3.351x10^{-6}$	$9.059x10^{-3}$
	12	$2.218x10^{-6}$	$7.622x10^{-4}$

MM – multiple myeloma, PCL – plasma cell leukemia

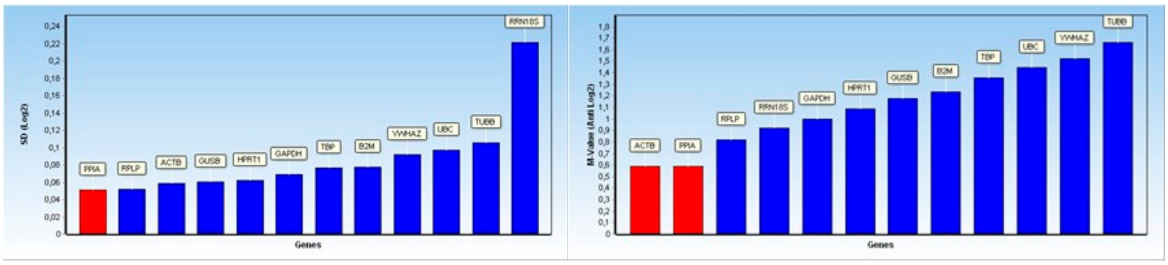

(**a**) (**b**)

Figure S1. Housekeeping gene analysis for normalization using TATAA Reference Gene Panel: (a) GeNorm software analysis. PPIA housekeeping gene with M = 0.586 and (b) NormFinder software analysis. PPIA housekeeping gene with SD = 0.0513.
